# Remember me - user-centered implementation of working memory architectures on an industrial robot

**DOI:** 10.3389/frobt.2023.1257690

**Published:** 2023-12-05

**Authors:** Jasmin Bernotat, Lorenzo Landolfi, Dario Pasquali, Alice Nardelli, Francesco Rea

**Affiliations:** ^1^ COgNiTive Architecture for Collaborative Technologies (CONTACT) Unit, Italian Institute of Technology (IIT), Genoa, Italy; ^2^ Department of Informatics, Bioengineering, Robotics and Systems Engineering (DIBRIS), University of Genoa, Genoa, Italy

**Keywords:** user-centered approach, evaluational studies, robot working memory, human-robot coexistence, robot navigation

## Abstract

The present research is innovative as we followed a user-centered approach to implement and train two working memory architectures on an industrial RB-KAIROS + robot: GRU, a state-of-the-art architecture, and WorkMATe, a biologically-inspired alternative. Although user-centered approaches are essential to create a comfortable and safe HRI, they are still rare in industrial settings. Closing this research gap, we conducted two online user studies with large heterogeneous samples. The major aim of these studies was to evaluate the RB-KAIROS + robot’s appearance, movements, and perceived memory functions before (User Study 1) and after the implementation and training of robot working memory (User Study 2). In User Study 1, we furthermore explored participants’ ideas about robot memory and what aspects of the robot’s movements participants found positive and what aspects they would change. The effects of participants’ demographic background and attitudes were controlled for. In User Study 1, participants’ overall evaluations of the robot were moderate. Participant age and negative attitudes toward robots led to more negative robot evaluations. According to exploratory analyses, these effects were driven by perceived low experience with robots. Participants expressed clear ideas of robot memory and precise suggestions for a safe, efficient, and comfortable robot navigation which are valuable for further research and development. In User Study 2, the implementation of WorkMATe and GRU led to more positive evaluations of perceived robot memory, but not of robot appearance and movements. Participants’ robot evaluations were driven by their positive views of robots. Our results demonstrate that considering potential users’ views can greatly contribute to an efficient and positively perceived robot navigation, while users’ experience with robots is crucial for a positive HRI.

## 1 Introduction

Amongst various contexts, robots have become an integral part of industrial working processes. The application of intelligent technologies to automate industrial processes and thereby to make them more efficient and safe was characteristic of Industry 4.0. Extending the principles of Industry 4.0, Industry 5.0 was marked by a transition to a strong user-centered perspective on technology use (see Psarommatis et al., 2023a; Azamfirei et al., 2023a; Konstantinidis et al., 2022b for an overview). Unlike ordinary industrial tools, robots are supposed to perform their given tasks autonomously in a shared environment with human workers. Therefore, with regard to Industry 5.0, developments in human-robot interaction (HRI) are increasingly driven by the idea of human-robot co-existence and collaboration. To enable smooth human-robot co-working, two perspectives need to be regarded. On the one hand, some technological requirements need to be met: Robots should be capable of localizing themselves with respect to their surroundings. To ensure a comfortable and safe HRI, robots need to adapt to the dynamics of a shared environment (see Konstantinidis et al., 2022a; Azamfirei et al., 2023a), which are determined by humans’ activities, intentions, and needs. On the other hand, the humans’ perspective needs to be taken into consideration: Robots should meet potential users’ expectations and needs to evoke positive perceptions (see Bernotat and Eyssel, 2017b; Bernotat and Eyssel, 2018).

Robot working memory architectures were found to be useful to achieve the technological requirements for an efficient and safe human-robot co-existence because they enable autonomous and environmental-aware robot actions (see, e.g., Landolfi et al. (2023); Reich et al. (2020); Joo et al. (2019); Jung et al. (2019)). Similar to human working memory (see Baddeley (2000; 2010)), robot working memory architectures enable a robot to store, organize, and process data. Using robot working memory architectures, a robot is supposed to ‘learn’ based on prior experiences, that is, to ‘decide’ what information to use in order to improve future behavior according to the dynamics of shared environments.

One state-of-the-art working memory architecture that is commonly used in machine learning is GRU (Gated Recurrent Unit, Cho et al. (2014)). GRU is a sort of recurrent neural network architecture (RNN) that is often used to process data sequences, such as language input (see Cho et al., 2014). Learning is enabled by iterative adjustments of internal parameters (see Cho et al., 2014; Landolfi et al., 2023). However, current state-of-the-art architectures were limited in controlling and prioritizing stored information as required to solve more complex tasks. Kruijne and others thus proposed WorkMATe (Working Memory Architecture for Task Execution), a biologically-inspired working memory architecture whose key components include a gated memory circuit driven by internal actions (Kruijne et al., 2021). More precisely, training occurs in a biologically-inspired manner based on attentional feedback and reward prediction errors. That is, the system optimizes its behavior based on reward feedback similar to biological dopamine-based processes that enable animals and humans to learn and adapt their behavior (see Glimcher, 2011; Wang et al., 2018). WorkMATe enables to store and process multiple inputs separately and to update and transfer trained adaptations to new contexts and stimuli. All this makes WorkMATe well suitable for complex memory tasks and allows for flexible and task-oriented memory control.

Overall, working memory architectures allow for flexible and task-oriented robot behavior when sensing humans and objects (e.g., leaving space, slowing down, stopping). These behavioral adaptations can make robot navigation safer and more predictable for humans (see Li et al., 2022; Landolfi et al., 2023). However, despite Industry 5.0’s strong focus on the humans’ perspective, the implementation of robot working memory was mainly regarded from the technological perspective. The humans’ perspective had been widely neglected so far.

Involving potential users’ perceptions and preferences already in the research and development processes of new technologies was found to lead to more positive user experiences and safety in human-technology interaction (see Mahmood et al., 2000; Ben Allouch et al., 2009; Schiffhauer et al., 2016; Bernotat and Eyssel, 2017a; Diehl et al., 2017; Robinson et al., 2020; Lacroix et al., 2023). Therefore, in the present research we addressed a research gap by putting the humans’ perspective into focus during the implementation and training of two working memory configurations on an industrial RB-KAIROS + robot (Robotnik (Valencia, Spain)): One working memory architecture was based on GRU (Cho et al., 2014) and the other was based on WorkMATe (Kruijne et al., 2021), the biologically-inspired alternative. Following a human-centered approach, we considered potential users’ ideas and perceptions of a comfortable and efficient human-robot co-existence right from the beginning of the implementation and training processes.

More precisely, we conducted two online user studies. The major aim of User Study 1 was to investigate participants’ judgements of the RB-KAIROS + robot’s appearance, movements, and perceived memory functions when presented in its initial state, i.e., with no working memory configuration implemented. In addition, we controlled for the effects of participants’ attitudes toward robots, social desirability, situational motivation to participate in the study, experience with technology and robots, and demographics on participants’ evaluations of the robot as covariates. The effects of these covariates were considered because they have been found to play a role in prior HRI research (Schiffhauer et al., 2016; Bernotat and Eyssel, 2017b; Bernotat et al., 2017; Meyer zu Borgsen et al., 2017; Bernotat and Eyssel, 2018; Bernotat et al., 2021). The consideration of the covariates therefore allowed for a more holistic view on the psychological aspects that might underlie participants’ perceptions of an industrial robot. To inspire further developments toward an efficient and positively perceived robot navigation, the secondary aim of User Study 1 was to explore participants’ ideas of robot memory in general and what aspects of the robot’s movements participants found positive and what aspects they would change. The expected benefit of exploring participants’ ideas of robot memory and their indications about the robot’s movements was to provide concrete practical implications for developers of robot memory and robot navigation.

User Study 2 served to evaluate the RB-KAIROS + robot’s appearance, movements, and perceived memory functions after the implementation and training of GRU and WorkMATe compared to no working memory in a between-subjects study. Analogous to User Study 1, the effects of participants’ attitudes, experience levels, social desirability, motivation to take part in the study, and demographics were controlled for (see Landolfi et al. (2023) for a technological evaluation of robot working memory that was done complementary). Moreover, using the same measures as in User Study 1 allowed us to validate our newly developed measures on robot appearance, robot movements, and robot memory. In this way, as an additional benefit of our research, we potentially offer suitable measures for future research on HRI in industrial settings that were lacking so far.

## 2 User study 1 - materials and methods

### 2.1 Participant sample

In total, *N* = 89 participants took part in User Study 1. However, *n* = 7 participants had to be excluded because they reported that they could not watch the video properly. From the remaining participants *n* = 82 participants, *n* = 40 completed the study in Italian language, *n* = 23 in German language, and *n* = 19 in English. Demographics consisted as follows:

**Age:** *M* = 37.91, *SD* = 15.66, range: 17–76 years; **gender:** female: *n* = 33; male: *n* = 29, non-binary: *n* = 3, undisclosed: *n* = 17; **nationality:** Italian: *n* = 30, German: *n* = 17, English-speaking country: *n* = 7 (i.e., Australia, United States, Canada, Great Britain), others: *n* = 10 (i.e., India, Nigeria, Mexico, France, Uzbek), undisclosed: *n* = 18; **native language:** Italian: *n* = 25, German: *n* = 21, English: *n* = 11, others: *n* = 7 (i.e., French, Hindi, Marathi, Spanish, Tamil, Telugu, Uzbek), undisclosed: *n* = 18; **professional status:** students: *n* = 23, employees: *n* = 29, others: *n* = 11 (i.e., retired, mother, unemployed), undisclosed: *n* = 19.

Overall, participants indicated fairly high levels of experience with technology in general, *M* = 5.31, *SD* = 1.54, but pretty low levels of experience with robots, *M* = 3.56, *SD* = 1.93. The majority of participants indicated to know robots mainly from media (e.g., books, movies): *n* = 44, followed from work: *n* = 30, other studies: *n* = 24, home: *n* = 15, and other contexts: *n* = 5 (i.e., research, medicine, school, traffic).

Only a minority of participants (*n* = 5) reported to have known the RB-KAIROS + robot from other studies before (not known from other studies: *n* = 59, undisclosed: *n* = 18). Likewise, *n* = 7 reported to have known the RB-KAIROS + robot from media (not known from the media: *n* = 57, undisclosed: *n* = 18).

All participants showed discrete response patterns on each of the measured constructs. That is, none of the participants responded to the questions following a specific pattern or showed extreme values, which might have suggested a lack of attention and motivation while completing the questionnaire.

### 2.2 Experimental procedure

**FIGURE 1:**
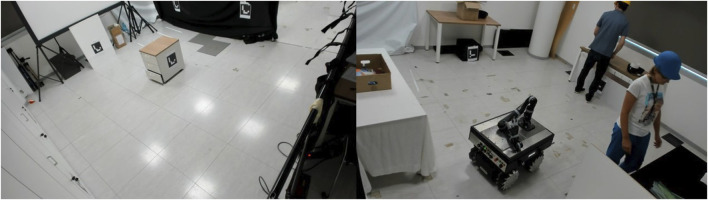
Scene of the video that shows the empty area (left) and the workers’ area (right) with the two workers and the RB-KAIROS + robot moving amongst them.

The study was conducted online using SurveyMonkey (San Matteo, California, USA), a tool for online studies. The online format was chosen because it was considered safer for participants. In addition, it allowed to easily recruit as large samples as possible across countries. The study was advertised on social media platforms, University mailing lists, and by friends and family in Italy, Germany, United Kingdom, and Australia. After giving consent to participate in the study, participants watched a short video sequence of about 2 minutes. The video sequence showed a working environment divided into two areas (see Figure 1). In one of the areas, the workers’ area, two alleged workers transported objects from one table to an opposite. In the other area, no people were around. It was thus also called the empty area. An RB-KAIROS + robot (Robotnik (Valencia, Spain), 2023, see Figure 1) moved between the two areas of the working environment according to a predefined sequence of way-points. The robot was shown in its initial state with no robot working memory configuration implemented. That is, the robot was not capable of performing behavioral adaptations based on the area or the presence of humans. It navigated with a maximum speed of 0.65 m/s and holonomic driving, using Time Elastic Band (TEB) (Rösmann et al., 2017) as local path planner. After having watched the video sequence, participants completed a questionnaire to evaluate the robot based on the scene they had watched. In total, participants needed about 15 min to complete the study.

### 2.3 Robot platform

The RB-KAIROS + robot (Robotnik Valencia, Spain, 2023); see Figure 1) is a rover robot built for industrial use (115Kg, 978 × 776 × 1.542 mm). It is supposed to navigate in empty halls as well as in shared environments with workers. For an efficient robot navigation, the RB-KAIROS + robot is equipped with four Ilon wheels that enable it to move holonomically, that is, in any direction. A frontal RGB-D camera (640 × 480 resolution, 30 fps) and a pair of SICK S300 laser scanners are installed to capture the environment. Enabling 360°vision, the laser scanners are placed at the top right and at the bottom left corner of the robot’s in 30 cm height. Furthermore, a Kinova Jaco^2^ assistive robotic arm is mounted on top of the robot’s base.

### 2.4 Questionnaire measures

For most questionnaire measures, 7-point Likert scales were used to capture participants’ agreement to an item’s content. High scores indicated high endorsement of a respective construct. Items were recoded if necessary. Indices were composed based on their Cronbach’s alphas (*α*) as a measure of the construct’s reliability (Cronbach, 1951). All measured constructs showed satisfying reliabilities, except for the situational motivation scale which had thus to be excluded from statistical analyses (see below for each scale’s Cronbach’s alpha, see Cronbach (1951); Schmitt (1996) for interpretations of Cronbach’s alpha).

**Robot Appearance:** The robot’s appearance was evaluated with eleven items that were adapted based on Bernotat and Eyssel’s Bernotat and Eyssel (2017a) previous research on robot design (e.g., “Judging by the appearance of the robot, I had the impression that it looked technically sophisticated.”, *α* = .69). One item (“Judging by the appearance of the robot, I had the impression that it looked like any technical tool that I would not have recognized as a robot.”) was removed from the index to increase the internal consistency of the scale.

**Robot Movements:** The robot’s movements were judged by using 17 self-generated items (e.g., “Watching the robot moving, I had the impression the robot’s movements were smooth.”, *α* = .83).

In addition, to consider potential users’ perceptions of robot memory and robot movements as potential memory-based visible outcomes, we posed three open-response questions on robot movements: 1. “Please describe in what direction the robot’s wheels moved.”, 2. “Please describe what you liked about the robot’s movements.”, 3. “Please describe what you would change about the robot’s movements.”. The first question served to enquire whether participants were aware of the holonomic direction of the robot’s wheels. The open-response format prevented us from forcing participants to make guesses about the robot’s driving. The latter two questions served to potentially improve the robot’s movements in the future.

**Robot Memory:** Participants perceptions of robot memory functions were assessed by 14 items (e.g., “Watching the robot, I had the impression the robot could autonomously decide whether to adapt its behavior to a specific situation or not.”, *α* = .91). The items were based on literature on trust, memory, and automation in humans and in robots (e.g., Baddeley, 2000; Parasuraman et al., 2000; Hancock et al., 2021), but adapted to fit the purpose of the present research.

In addition, an open-response format was used to enquire participants’ imagination of robot memory (“What do you think would characterize robot memory?”).

**Positive and Negative Attitudes Toward Robots:** Participants’ positive and negative attitudes toward robots were assessed with 10 items each (positive attitudes: e.g., “I think robots are great innovations.”, *α* = .76, negative attitudes: e.g., “I would feel uneasy if robots really had emotions.”, *α* = .77). The negative attitudes toward robots sub-scale was an adapted and extended version of Nomura and colleagues’ scale Nomura et al. (2006). Nomura and colleagues did not provide a sub-scale on positive attitudes toward robots. Therefore, items on positive attitudes were self-generated to assess the full spectrum of attitudes toward robots.

**Social Desirability:** 17 items by Stöber (German version: Stöber (1999), English version; Stöber (2001), e. g., “In traffic I am always polite and considerate of others.”, *α* = .78) were used to measure participants’ desire to behave in a socially expected manner.

**Situational Motivation:** With four items by Guay and colleagues Guay et al. (2000), we assessed participants’ motivation to participate in the present study (e.g., “By personal decision.”, *α* = .45).

**Experience With Technology and Robots:** With one item each, we enquired participants’ experience with technology in general (“Please indicate to what extent you have experience with technology in general.”) and with robots in particular (“Please indicate to what extent you have experience with robots.”). In addition, to deepen insights into participants’ experience with robots, we used a multiple choice format to assess from what context participants mainly knew robots from (response options: media (e.g., movies, books), work, home, other studies, and other contexts which had to be specified). In addition, participants were asked to indicate whether they knew the RB-KAIROS + robot before from other studies or media.

**Manipulation Check:** We assessed whether participants could watch the video properly with both image and sound.

## 3 User study 1 - results

### 3.1 Participants’ evaluations of the RB-KAIROS + robot

To investigate participants’ evaluations of the RB-KAIROS + robot when displayed in its initial state without robot memory implemented (see Section 2.2), a multivariate analysis of covariance (MANCOVA) was conducted. Participants’ evaluations of robot appearance, robot movements, and perceived robot memory were considered as dependent measures. Participants’ positive and negative attitudes toward robots, social desirability, experience with technology and robots, and demographics (i.e., sample language, participant age, gender, nationality, native language, and professional status) were considered as covariates in order to control for their effects on participants’ evaluations of the robot. To enable a valid interpretation of *p*-values, effect sizes (*η* *p* ^2^) and statistical power (1-*β*) were reported complementary. Statistically significant effects of the covariates on the dependent measures were confirmed by applying the principle of parsimony (see Tabachnick and Fidell, 2007). Pearson correlations between the dependent measures and the covariates that had statistically significant effects on the dependent measures were performed to investigate the role of the covariates in more detail.

**FIGURE 2:**
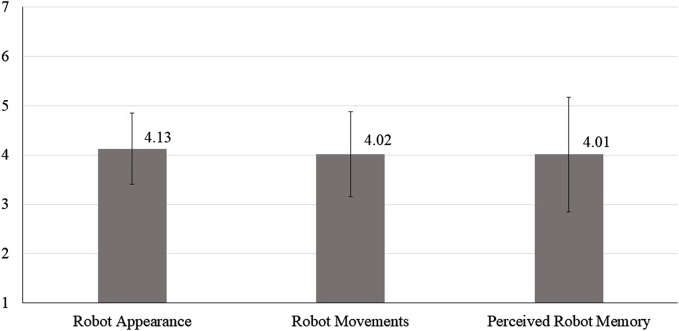
Mean scores (grey bars) and standard deviations (soft lines) of participants’ evaluations of robot appearance, robot movements, and perceived robot memory.

Inspecting participants’ mean scores on robot appearance, robot movements, and perceived robot memory, participants’ evaluations of the robot were moderate (see Figure 2).

Regarding the covariates’ effects, participants’ negative attitudes toward robots statistically significantly affected their evaluations of robot appearance, *F* (1,48) = 4.53, *p* = .038, *η* *p* ^2^ = .086, 1-*β* = .550. Participant age statistically significantly affected participants’ evaluations of perceived robot memory, *F* (1,48) = 4.78, *p* = .034, *η* *p* ^2^ = .091, 1-*β* = .572. The effects of the remaining covariates on the dependent measures were not statistically significant (*p*s > .05). Following the principle of parsimony (see Tabachnick and Fidell, 2007), the same analysis was performed again with only participant age and negative attitudes toward robots as covariates in order to confirm the effects.

The statistically significant effect of participants’ negative attitudes toward robots on their evaluations of robot appearance was confirmed, *F* (1,58) = 9.95, *p* = .003, *η* *p* ^2^ = .146, 1-*β* = .873. The same accounted for the statistically significant effect of participant age on participants’ evaluations of perceived robot memory, *F* (1,58) = 6.31, *p* = .015, *η* *p* ^2^ = .098, 1-*β* = .695. Furthermore, the effect of participant age on their evaluations of robot movements turned out statistically significant, *F* (1,58) = 4.12, *p* = .047, *η* *p* ^2^ = .066, 1-*β* = .515.

Pearson correlations showed the more negative attitudes participants shared, the less positive their judgements of robot appearance, *r* (60) = −.42, *p* < .001. The older participants, the less positive their evaluations of perceived robot memory functions, *r* (62) = .-.39, *p* = .002, and robot movements, *r* (62) = −.30, *p* = .017.

Exploratory analyses were performed to investigate the relation between participant age, negative attitudes toward robots, and participants’ evaluations of the robot in more detail (see Section 3.4).

### 3.2 Participants’ ideas of robot memory

Participants’ written descriptions of robot memory were analyzed to answer our secondary research question of what participants imagined being characteristic of robot memory (see Section 1). More precisely, to quantify participants’ responses, we counted the frequencies of the words that participants used to describe their subjective ideas of robot memory. Words were then clustered according to their frequencies in participants’ descriptions. That way, six different aspects of robot memory were identified, namely, memory and recall, learning and adaptation, hard- and software components, data collection and information storage, advantages of robot memory, and risks of robot memory (see Table 1):

**TABLE 1:** Aspects of robot memory based on word frequencies in participants’ subjective descriptions of robot memory.

Aspects of robot memory	Examples from participants’ descriptions	Word frequency
Memory and Recall	Memory, remember, recall, reuse, retrieve, process, represent information	24
Learning and Adaptation	Learning, evaluation, adaptation, repetition, avoiding mistakes and limitations, improving, deciding what information to use, intelligence	19
Hard- and Software Components	Hardware, software, sensors, processor, computer, storage device, lidar sensors, codes, neural networks, machine learning, deep learning, algorithms (i.e., face recognition, motion profile)	18
Data Collection and Information Storage	Capture, collect, and acquire data, extract, save, store, information accordingly	17
Advantages of Robot Memory	Robot memory based on human memory can facilitate human-robot interaction, enable communication between robots, and overcome human weaknesses and limitations (e.g., making errors, forgetting, being affected by external factors, subjective judgments, emotions)	12
Risks of Robot Memory	Robot memory can get lost in an instant, can be controlled, manipulated, or changed by an external controller	2

Words related to **memory and recall** of information occurred most frequently, followed by terms referring to **learning and adaptation** based on prior information. Learning and adaptation were closely related to the notion of ‘intelligence’ and the robot’s ability to evaluate past experiences and to ‘decide’ what information to use to overcome limitations and errors. **Hard- and software components** were considered crucial for robot memory as they enable to collect and process a variety of data, such as face recognition and motion profiles. In this context, robot memory was linked to **data collection and information storage**. Participants also mentioned potential **advantages of robot memory** and potential **risks of robot memory**. According to word frequencies, the potential advantages of robot memory out-weighted perceived risks. Robotic memory has been described as a way to make HRI easier, smoother, and more reliable because robotic memory could overcome human weaknesses and limitations such as human error, forgetting, and the effects of subjective judgments and emotions. Some participants even felt that robot memory could enable communication between robots. Only a few risks of robot memory were mentioned, such as the concern robot memory could be lost or controlled and manipulated by an external operator.

### 3.3 Suggestions for improvement of robot movements

To potentially inspire future developments of robot movements, participants were asked to list aspects they liked about the robot’s movements and aspects they would change. In line with participants’ moderate evaluations of robot movements (see Figure 2), the robot’s movements were appreciated and criticized at the same time. Participants’ responses can be summed as follows:

Some participants appreciated that the robot was moving smoothly, noiselessly, with an adequate velocity, and that it stopped or changed its direction when detecting humans or objects. This was described as making the robot’s movements predictable, precise, and trustworthy. One participant even had the impression that the robot would have tried to move with the same speed as the workers. This was definitively not the case because behavioral adaptations of the robot’s speed based on robot working memory were not yet implemented. About a third of the participants noticed the robot’s holonomic driving (holonomic driving detected: *n* = 32; not detected: *n* = 30, undisclosed: *n* = 20). Some of the participants who noticed the holonomic driving found it interesting and efficient. Others found the holonomic driving was unexpected and would make the robot’s movements unpredictable and irritating. Perceptions of unpredictability and irritation were strengthened because the robot’s wheels did not turn left or right when the robot’s driving direction changed and the robot’s intention to move was not clear. As such, some participants criticized the robot for crossing the workers’ path too often and approaching the workers too closely and too fastly without any obvious reason. To make the robot’s movements smoother and more predictable, participants suggested to let the robot drive non-holonomically, to increase the robot’s distance to humans and objectives, to adapt its speed and trajectory dependent of human presence, and to indicate the robot’s intention and changes in its motion. Participants moreover stated the robot should be able to anticipate the workers’ actions.

### 3.4 Exploratory analyses

Investigating participants’ evaluations of the robot, participant’s negative attitudes toward robots and age had statistically significant effects on their overall judgments of the robot (see Section 3.1).

Previous literature showed that older participants feel less certain in dealing with technology and robots (Damant and Knapp, 2015; Goodman-Deane et al., 2020; Robinson et al., 2020). Confirmatively, particularly some older participants stated that they did not feel competent to judge a robot because they felt they had too little experience with robots. Participants’ levels of experience with technology and robots, in turn, were found to be decisive for their attitudes toward robots and technology (see Connolly et al., 2020; Gjelaj et al., 2020). Therefore, exploratory analyses were performed to investigate the relationship between participants’ evaluations of the robot, participant age, and participants’ attitudes toward robots in more detail.

To do so, a MANCOVA was performed with participants’ experience with technology and experience with robots while the effects of participant age were controlled for. Participant age statistically significantly determined participants’ experience with technology, *F* (1,62) = 18.55, *p* < .001, *η* *p* ^2^ = .230, 1-*β* = .989, and experience with robots, *F* (1,62) = 16.51, *p* < .001, *η* *p* ^2^ = .210, 1-*β* = .979. Pearson correlations showed the older participants, the lesser experience with technology, *r* (62) = −.48, *p* < .001, and experience with robots, *r* (62) = −.46, *p* < .001, they reported.

**FIGURE 3:**
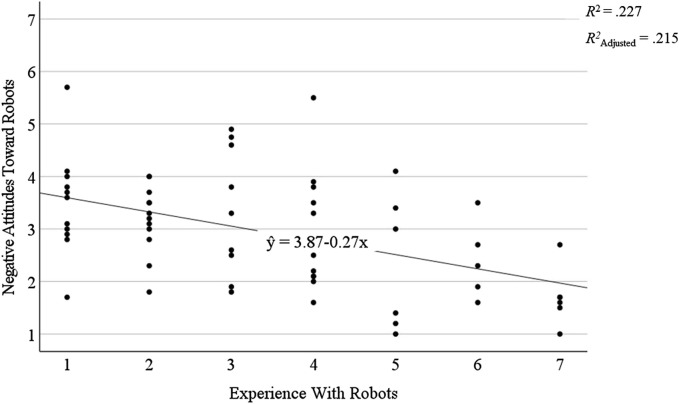
Graphical depiction of the linear regression model between participants’ negative attitudes toward robots as a dependent variable and their experience with robots as a predictor as well as *R* ^2^, *R* ^2^_Adjusted_, and the underlying regression equation.

Confirming previous research (Damant and Knapp, 2015; Goodman-Deane et al., 2020; Robinson et al., 2020), the results of this first exploratory analysis showed that participant age was linked to lower levels of experience with technology. What however still remains unclear is whether the low levels of experience with technology and robots or participant age *per se* led to more negative attitudes toward robots. To address this question, we performed a linear regression analysis with participant age, experience with technology, and experience with robots step-wise entered as predictors of participants’ negative attitudes toward robots. The overall model was statistically significant, *F* (1,60) = 17.67, *p* < .001, and accounted for about 22% of the variance, *R* ^2^ = .227, *R* ^2^_Adjusted_ = .215. The only statistically significant predictor of participants’ negative attitudes toward robots was participants’ experience with robots, *β* = −.477, 95%-CI [-0.40; −0.14], *p* < .001. Participants’ experience with technology, *β* = −.273, *p* = .069, and participant age, *β* = .141, *p* = .273, did not statistically significantly predict participants’ negative attitudes toward robots and were thus excluded from the overall model. As indicated by the negative *β* weight which corresponds to the Pearson correlation, *r* (60) = −.48, *p* < .001, participants reported less negative attitudes toward robots, the more experience with robots they shared (see also Figure 3). Regarding participants’ mean scores, participants’ negative attitudes toward robots were relatively low, but with a quite large standard deviation indicating large differences across participants, *M* = 2.94, *SD* = 1.07, compared to their mean scores on positive attitudes toward robots, *M* = 5.66, *SD* = 0.80.

Taken together, the results of these exploratory analyses indicate that not participant age *per se*, but participants’ low levels of experience with robots, resulted in more negative attitudes toward robots.

## 4 User study 2 - materials and methods

### 4.1 Participant sample

Addressing lay people and professionals of all ages and social backgrounds in Italy and Germany, *N* = 139 completed the study. Due to technical issues with the online survey platform and problems displaying the video properly, however, *n* = 45 participants had to be excluded from data analysis. From the remaining *n* = 94 participants, *n* = 46 completed User Study 2 in Italian language while *n* = 48 completed the German language version of the study. Demographics were as follows:

**Age:** *M* = 40.28, *SD* = 17.10, range: 13–77 years; **gender:** female: *n* = 57, male: *n* = 22, prefer not to answer: *n*: 2, undisclosed: *n* = 13; **nationality:** Italian: *n* = 36, German: *n* = 44, undisclosed: *n* = 14; **native language:** Italian: *n* = 36, German: *n* = 42, others: *n* = 2 (i.e., Irish, Swedish), undisclosed: *n* = 14; **professional status:** students: *n* = 24, professionals: *n* = 34 (i.e., architect, teacher, self-employed, employee, researcher), others: *n* = 21 (i.e., retired, searching for a job), undisclosed: *n* = 15.

Overall, participants reported moderate levels of experience with technology in general, *M* = 4.76, *SD* = 1.68, but low levels of experience with robots, *M* = 2.76, *SD* = 1.96. The majority of participants reported to know robots mainly from media (e.g., movies, books): *n* = 54, followed from the work: *n* = 33 and home context: *n* = 30, other studies: *n* = 26, and other contexts: *n* = 20 (i.e., friends, automotive industry, autonomous driving, space exploration, industry, factory work, trade shows, restaurants, University).

Only a minority of participants had known the RB-KAIROS + robot before: *n* = 18 reported to have known the RB-KAIROS + robot from previous studies (not known from other studies before: *n* = 62, undisclosed: *n* = 14), while *n* = 12 had known the RB-KAIROS+ from media (not known from media before: *n* = 68, undisclosed: *n* = 14). All participants showed indiscrete response patterns. That is, none of the participants responded to the questions following a specific pattern or showed extreme values which might have indicated a lack of attention and motivation while completing the questionnaire. Likewise, none of the participants indicated technical issues when watching the video.

### 4.2 Experimental design

In order to test the effects of robot working memory on participants’ evaluations of the RB-KAIROS + robot (see Section 1), a one factorial (robot working memory: WorkMATe vs GRU vs no working memory) - between-subjects design was realized (see AsPredicted #125198).

### 4.3 Experimental procedure

To reach an as large and heterogeneous sample as possible, User Study 2 was conducted online using SurveyMonkey (San Matteo, California, United States), and later, due to technical issues, SoSciSurvey (SoSci Survey GmbH, Munich, Germany). The study was advertised on social media platforms and by friends and family in Italy and Germany. To assure a high comparability between the results of User Study 1 and User Study 2, the experimental procedure was kept as similar as possible to User Study 1. First, participants watched a short video sequence of about 2 min which showed the RB-KAIROS + robot navigating between a workers’ area in which two workers performed a pick-and-place task and an empty area (see Figure 1). Unlike User Study 1, the robot was either shown with GRU, WorkMATe, or no working memory configuration, an adaptation needed to answer the underlying research question of User Study 2 whether the implementation and training of robot working memory would lead to more positive evaluations of the robot (see also Section 1). After having watched the video sequence, participants completed a questionnaire. In total, participants needed about 15 min to complete the study.

### 4.4 Working memory configurations

The robot navigated between the workers’ area and the empty area with either a GRU, WorkMATe, or no working memory configuration implemented (see Section 1, see also Landolfi et al. (2023) for more details on the implementation and training processes). During robot navigation, the working memory configurations, respectively the absence of robot working memory were reflected as follows:

When equipped with either GRU or WorkMATe working memory configurations, the robot adapted its behavior depending on whether it navigated in the workers’ area or in the empty area (*spacial awareness*) and depending on whether humans were around or not (*human awareness*). As soon as the robot sensed to be in the workers’ area in which humans could be around or as soon as it perceived humans, it slowed down from 0.65 m/s to 0.32 m/s and turned from holonomic to non-holonomic navigation. As the RB-KAIROS + robot is supposed to be used in large industrial halls, the robot could be aware of being in the empty area while in fact no people were around. In such a case, the RB-KAIROS + robot would slow down to 0.32 m/s while remaining holonomic driving. As in our studies, workers were around in their supposed area all the time, this did however not happen so that the robot commonly slowed down and turned to non-holonomical driving in the workers’ area. These adaptations of robot navigation were well in line with participants’ suggestions for robot adaptation (see Section 3.3); they were thus supposed to increase positive user experiences by making robot navigation safer and more predictable. Enabling bio-inspired learning and behavior adaptation, the robot was positively rewarded for showing correct behavior adaptations and negatively rewarded for wrong behavior adaptations when equipped with WorkMATe (see also Landolfi et al., 2023). When no working memory configuration was implemented, no adaptations in terms of speed and holonomicity were performed during robot navigation.

### 4.5 Questionnaire measures

The same questionnaire was used as in User Study 1 (see Section 2.4). The only adaptation of the questionnaire was that participants’ ideas about robot memory and their suggestions for improving robot movements were not enquired because these aspects were of particular interest in User Study 1 to inspire future developments of robot working memory.

Analogous to User Study 1, indices of the measured constructs were calculated based on internal consistencies (Cronbach’s alpha) with high scores indicating high endorsement of the measured construct (see Section 2.4). Due to a too low internal consistency (*α* = .12), the situational motivation scale had to be excluded from statistical analyses. For all other constructs, internal consistencies were moderate to high: **Robot appearance:** *α* = .67 (after excluding the item ‘Judging by the appearance of the robot, I had the impression that it looked like any technical tool that I would not have recognized as a robot.’), **robot movements:** *α* = .87, **perceived robot memory:** *α* = .92, **positive attitudes toward robots:** *α* = .90, **negative attitudes toward robots:** *α* = .87, **social desirability:** *α* = .81.

## 5 User study 2 - results

### 5.1 Participants’ evaluations of the RB-KAIROS + robot

To answer the research question whether participants judged the robot’s appearance, movements, and perceived memory functions differently depending of the robot’s working memory configuration (GRU, WorkMATe, and no working memory, see Section 1), a multivariate analysis of covariance (MANCOVA) was performed similar to User Study 1 (see Section 3.1). Participants’ evaluations of robot appearance, robot movements, and perceived memory functions, the dependent measures, were investigated as a function of robot working memory configuration (GRU, WorkMATe, and no working memory). Analogous to User Study 1, participants’ positive and negative attitudes toward robots, social desirability, experience with technology and with robots, and demographics (i.e., sample language, participant age, gender, nationality, native language, professional status) were considered as covariates in order to control for their effects on participants’ evaluations of the robot. For a valid interpretation of *p*-values, effect sizes (*η* *p* ^2^) and statistical power (1-*β*) were reported complementary. Statistically significant effects of the experimental condition (GRU, WorkMATe, and no working memory) and the covariates were confirmed following the principle of parsimony (see Tabachnick and Fidell, 2007). Pearson correlations between the dependent measures and the covariates that had statistically significant effects on the dependent measures were performed to investigate the role of the covariates in more detail.

The experimental condition statistically significantly affected participants’ evaluations of perceived robot memory functions, *F* (2,67) = 3.21, *p* = .047, *η* *p* ^2^ = .087, 1-*β* = .595, but not their evaluations of robot appearance, *F* (2,67) = 0.10, *p* = .904, *η* *p* ^2^ = .003, 1-*β* = .065, and robot movements, *F* (2,67) = 1.23, *p* = .300, *η* *p* ^2^ = .035, 1-*β* = .259.

Regarding the covariates, participants’ endorsement of positive attitudes toward robots had statistically significant effects on participants’ evaluations of robot memory functions, *F* (1,67) = 14.07, *p* < .001, *η* *p* ^2^ = .174, 1-*β* = .959. The effects of the remaining covariates on participants’ evaluations of the robot were not statistically significant (*p*s > .05).

Following the principle of parsimony (see Tabachnick and Fidell, 2007), the same analysis was run again, but with all covariates removed except for positive attitudes toward robots (see Tabachnick and Fidell, 2007).

**FIGURE 4:**
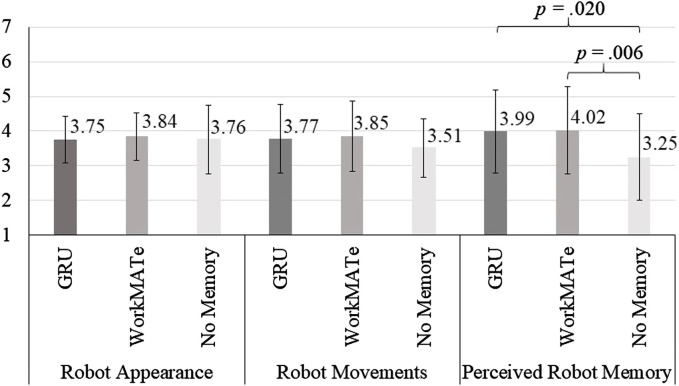
Mean scores (grey bars) and standard deviations (soft lines) of participants’ evaluations of robot appearance, robot memory, and perceived robot memory per experimental condition and *p*-values indicating statistically significant differences between experimental conditions.

In the more parsimonious model, the statistically significant effect of the experimental condition on participants’ evaluations of perceived robot memory functions was confirmed, *F* (2,77) = 4.29, *p* = .017, *η* *p* ^2^ = .100, 1-*β* = .732. Pairwise comparisons showed that the difference between WorkMATe and no working memory was statistically significant, *M*_Difference_ = 0.85, *SE* = 0.30, *p* = .006, 95%-CI [0.25; 1.45]. The same accounted for the difference between GRU and no working memory, *M*_Difference_ = 0.72, *SE* = 0.30, *p* = .020, 95%-CI [0.12; 1.32], while evaluations of perceived robot memory functions between WorkMATe and GRU were not statistically significant, *M*_Difference_ = 0.14, *SE* = 0.26, *p* = .609, 95%-CI [-0.39; 0.66] (see Figure 4). That is, the implementation of WorkMATe and GRU working memory configurations both resulted in more positive perceptions of robot memory functions.

The effects of the experimental condition of participants’ evaluations of robot appearance, *F* (2,77) = 0.29, *p* = .751, *η* *p* ^2^ = .007, 1-*β* = .094, and robot movements, *F* (2,77) = 1.28, *p* = .283, *η* *p* ^2^ = .032, 1-*β* = .270, did not turn out statistically significant. That is, the implementation of WorkMATe and GRU working memory did not statistically significantly affect participants’ evaluations of robot appearance and robot movements (see also 4 for graphical depictions of participants’ evaluations of the robot).

However, the statistically significant effect of positive attitudes toward robots on participants’ evaluation of robot memory functions was confirmed, *F* (1,77) = 34.71, *p* < .001, *η* *p* ^2^ = .311, 1-*β* = 1.00. Furthermore, the effects of positive attitudes toward robots on participants’ evaluations of robot appearance, *F* (1,77) = 10.97, *p* = .001, *η* *p* ^2^ = .125, 1-*β* = .905, and robot movements, *F* (1,77) = 24.99, *p* < .001, *η* *p* ^2^ = .245, 1-*β* = .999. Effect sizes and statistical power can be considered sufficiently high.

Pearson correlations showed the more positive attitudes toward robots participants shared, the more positive their evaluations of robot appearance, *r* (79) = .35, *p* = .001, robot movements, *r* (79) = .48, *p* < .001, and perceived robot memory functions, *r* (79) = .53, *p* < .001. Overall, participants shared rather positive attitudes toward robots, *M* = 5.31, *SD* = 1.10, than negative attitudes toward robots *M* = 3.64, *SD* = 1.40.

### 5.2 Exploratory analyses

**FIGURE 5:**
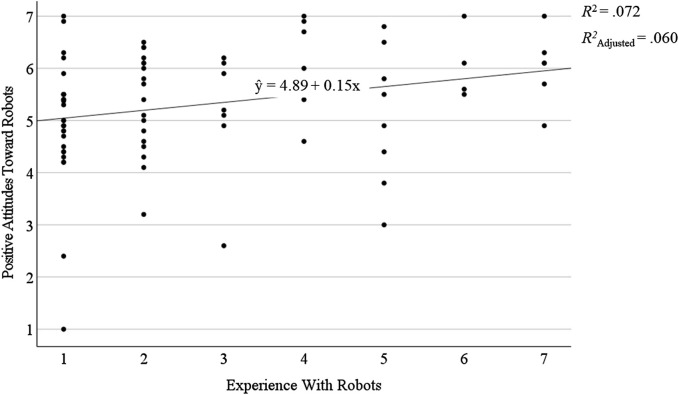
Graphical depiction of the linear regression model between participants’ positive attitudes toward robots as a dependent variable and their experience with robots as a predictor as well as *R* ^2^, *R* ^2^_Adjusted_, and the underlying regression equation.

In line with previous research (see Connolly et al., 2020; Gjelaj et al., 2020), participants’ experience with robots determined their attitudes toward robots in User Study 1 (see Section 3.4). Therefore, we explored whether participants’ experience with robots and experience with technology had also affected their positive attitudes in User Study 2. To do so, a linear regression was performed analogous to User Study 1. Participants’ experience with robots and experience with technology were entered step-wise as predictors of participants’ positive attitudes toward robots. The overall model was statistically significant, *F* (1,78) = 6.07, *p* = .016, and accounted about 6% of the variance, *R* ^2^ = .072, *R* ^2^_Adjusted_ = .060. Participants’ experience with robots was the only statistically significant predictor of participants’ positive attitudes toward robots, *β* = .269, *p* = .016, 95%-CI [0.03; 0.27]. Participants’ experience with technology was excluded from the overall model because it was not a statistically significant predictor of participants’ positive attitudes toward robots, *β* = .099, *p* = .468. As indicated by the positive *β* weight which corresponds to the Pearson correlation, participants’ positive attitudes toward robots increased with higher levels of experience with robots, *r* (78) = .27, *p* = .008. The relation between participants’ positive attitudes toward robots and their experience with robots is displayed in Figure 5. Overall, participants’ experience with robots was fairly low, *M* = 2.76, *SD* = 1.96, compared to their levels of experience with technology, *M* = 4.76, *SD* = 1.68 (see also Section 4.1).

## 6 General discussion

In the presented research, we followed a user-centered approach while implementing and training two working memory architectures on an industrial RB-KAIROS + robot (Robotnik Valencia, Spain, 2023): One of these architectures was based on GRU (Cho et al., 2014), a commonly used state-of-the-art architecture, the other was based on WorkMATe (Kruijne et al., 2021), a biologically-inspired alternative. Emphasizing the humans’ perspective on HRI, we considered potential users’ ideas and perceptions of robot navigation already before initiating the implementation and training processes as recommended by previous research (e.g., Mahmood et al., 2000; Ben Allouch et al., 2009; Schiffhauer et al., 2016; Bernotat and Eyssel, 2017a; Diehl et al., 2017; Azamfirei et al., 2023b; Psarommatis et al., 2023b; Lacroix et al., 2023). This approach was innovative because, so far, only little attention has been paid to the humans’ perspective when robot working memory was implemented and trained. More precisely, we conducted two online user studies in which we tested large and heterogeneous samples of Italian, German, and in User Study 1 also English-speaking participants of all ages and professional backgrounds. To identify potential biases, the effects of participants’ attitudes, prior experience with robots and technology, social desirability, and demographics on participants’ evaluations of the robot were controlled for (see Bernotat and Eyssel, 2017a; Bernotat et al., 2017; Meyer zu Borgsen et al., 2017; Bernotat et al., 2021). Moreover, in order to assess participants’ overall judgments of the robot as well as their individual ideas and preferences for robot navigation, quantitative measures and qualitative open response formats were used complementary. In User Study 1, we primarily investigated participants’ evaluations of the RB-KAIROS + robot in terms of appearance, movements, and perceived memory functions when the robot was presented with no robot working memory implemented. To enable potential future adaptations of robot behavior that might lead to positive user experiences, we furthermore assessed participants’ ideas of robot memory and asked what aspects of the robot’s movements participants found positive and what aspects they would change. GRU and WorkMATe architectures were then implemented and trained accordingly. User Study 2 was conducted after the implementation and training processes. The major aim of User Study 2 was to evaluate participants’ perceptions of robot appearance, movements, and perceived memory functions comparing WorkMATe and GRU against the initial state of no working memory in a between-subjects study.

In User Study 1, mean evaluations of the robot’s appearance, movements, and perceived memory functions ranged around scale midpoint indicating neither particularly positive, nor negative evaluations. Participants’ age had statistically significantly affected their evaluations of the robot’s memory and movements: The older participants were, the less positively they judged the robot’s memory and the robot’s movements. Moreover, participants negative attitudes toward robots resulted in less positive evaluations of the robot’s appearance. Exploratory analyses revealed a statistically significant relationship between participants’ age and their experience with technology and robots. The older participants, the lower levels of experience with technology and robots they shared which is well in line with previous research (Damant and Knapp, 2015; Goodman-Deane et al., 2020; Robinson et al., 2020). However, results of a linear regression analysis showed that not participant age *per se*, but participants’ experience with robots was decisive for their negative attitudes toward robots. Independent of participant age, less negative attitudes toward robots were predicted by higher levels of experience with robots.

The evaluation of a robot’s movements and memory requires a certain understanding of a robot’s functionality. As lower levels of experience with robots were linked to increased age, it seems plausible that participant age had affected participants’ evaluations of the robot’s movements and memory functions in User Study 1. This might be confirmed by the fact that particularly elderly participants reported they had felt uncertain how to judge a robot because they felt too in-experienced with robots. However, the effect of participants’ experience with robots on their negative attitudes toward robots that was found in User Study 1 suggests that perceived low experience with robots might have evoked feelings of uncertainty how to evaluate a robot independent of participant age. In interpersonal contact situations, uncertainty was found to lead to negative emotions and a perceived lack of control (Grieve and Hogg, 1999; Stewart et al., 2019). A relationship between a perceived lack of experience and a perceived lack of control in HRI seems plausible given that the negative attitudes toward robots scale included items that are closely related to the fear of robots taking control, e.g., “I fear that in the future society will be dominated by robots.”

Complementary to quantitative evaluations of the RB-KAIROS + robot, participants in User Study 1 provided fruitful insights about their general ideas of robot memory and made concrete suggestions for the improvement of robot movements. Clustering words that participants had used to describe robot memory by frequencies, robot memory was found to comprise six core aspects: memory and recall, learning and adaptation, hard- and software components, data collection and information storage, potential advantages and potential risks of robot memory. Regarding the potential advantages of robot memory, participants described that robot memory might facilitate communication between humans and robots and that it might help to overcome human weaknesses. Robot memory was furthermore seen as a chance to enable communication between robots. A potential risk associated with robot memory was that data could get lost. More importantly, confirming the assumption that a perceived loss of control over HRI was one of participants’ concerns, a feared risk was that robot memory could be controlled or manipulated by an external party (see Table 1). However, in line with participants’ overall rather positive attitudes toward robots, more advantages of robot memory were described than potential risks. In line with participants’ moderate evaluations of robot movements on the explicit measures, positive aspects about robot movements and suggestions for improvement were balanced. About a third of the participants noticed the robot’s holonomic driving. Some participants appreciated the holonomic driving as being interesting, innovative, and as making robot navigation more efficient. Other participants criticized the holonomic driving as being confusing and making robot navigation unpredictable. Feelings of confusion and a lack of predictability were strengthened because the RB-KAIROS + robot did not provide any cues to indicate its moving direction and intention. Participants thus suggested to implement holonomic driving and adaptations of the robot’s velocity dependent of human presence. Participants desired to have clear indicators of robot moving direction and intention. They felt this would make robot navigation safer, more predictable, and thus more comfortable.

User Study 2 followed after working memory architectures based on GRU and WorkMATe were implemented. In line with participants’ suggestions for the improvement of robot navigation in User Study 1, robot working memory enabled adaptations of holonomic driving and velocity dependent of human presence (see Section 4.4). Analogous to User Study 1, participants’ evaluations of robot appearance, movements, and perceived memory functions were assessed. Exceeding User Study 1, however, the robot was evaluated in a between-subjects study when it was shown with WorkMATe, GRU, or no working memory implementation. Similar to User Study 1, participants’ overall evaluations of the robot were moderate. Evaluations of robot appearance and robot movements did not statistically significantly differ between GRU, WorkMATe, and the no working memory condition. However, there was a statistically significant effect of the experimental condition on participants’ evaluations of robot working memory. Adaptations of robot navigation based on WorkMATe and GRU working memory led to more positive perceptions of robot memory functions than no working memory implementation. This finding complements results of our technological evaluation of the robot working memory implementations: We found a clear advantage of WorkMATe and GRU over no robot working memory in terms of safety and energy consumption during robot navigation (see Landolfi et al., 2023). Taken together, the results of User Study 2 suggest that participants well noticed improvements in robot navigation due to robot working memory. This accounted for GRU, the current state-of-the-art, and for WorkMATe, the biologically inspired alternative alike. Moreover, in User Study 2, participants’ evaluations of robot appearance, movements, and perceived memory functions were driven by their positive attitudes toward robots. Positive views of robots resulted in more positive evaluations of the robot on all three measures. Exploratory analyses showed that participants’ experience with robots was a predictor of their positive attitudes toward robots. This confirms findings of User Study 1 that participants’ experience with robots is decisive for positive views of robots.

Remarkably, in both user studies, participants shared fairly low levels of experience with robots, but a good command of technology use. Most participants reported to know robots mainly from media (see also Bernotat et al. (2017); Bernotat et al. (2021) for confirming findings) in which robots are represented as threatening on the one hand and as useful on the other hand. Despite some resentments against robots, participants shared fairly positive attitudes toward robots reflecting views of robots as useful innovations. This, in turn, suggests an overall readiness to accept robots if participants know how to use them in a beneficial way. This would be in line with research by Robinson and colleagues (Robinson et al., 2020) who found that participants’ willingness to use a robot, their self-efficacy in HRI, and the robot’s likeability were increased after a training on how to use a social robot and an interaction with it. Amongst the robot’s likeability, participants’ self-efficacy, that is, their belief in their ability to use and control robot use (see also Bandura, 1977; Schwarzer and Jerusalem, 1995; Pütten and Bock, 2018), crucially determined their willingness to interact with the robot in the future.

### 6.1 Limitations and implications for future research

With increased robot release more lay people will interact with robots in several contexts reaching from work to private areas. Robots, in turn, become increasingly autonomous social agents and co-workers instead of being mere tools. Therefore, as our results of User Study 1 and User Study 2 clearly indicate, the effects of participants’ experience with robots on their attitudes and emotions toward robots should be taken into sharper focus of future HRI research and development. Our results further suggest that a more detailed view of participants’ experience with robots as a multi-dimensional construct is needed. Therefore, Neyer and colleagues’ scale of technology commitment (see Neyer et al., 2012) consisting of perceived control over technology, perceived competence in its use, and acceptance of technology could be applied to HRI. Likewise, Rosenthal-von-der-Pütten and Bock’s self-efficacy in HRI scale (Pütten and Bock, 2018) might be suitable in future HRI research. This way, the assumption that was derived from our findings could be tested that uncertainty how to use a robot might be linked to a perceived lack of control in HRI which, in turn, is supposed to determine participants’ attitudes toward robots. This could help to encounter the cause for possible resentments against robots and thus to enhance acceptance and comfort of HRI in shared environments. To really grasp the dynamics that underlie an efficient and comfortable HRI in various settings, however, we recommend a more detailed consideration of various groups of potential users (see also Azamfirei et al., 2023b; Psarommatis et al., 2023b). To illustrate, in our research, we opted for heterogeneous samples to avoid possible biases and to reflect a general view on HRI with an industrial robot that is equipped with human-aware robot navigation. However, in line with previous research (see Damant and Knapp, 2015; Goodman-Deane et al., 2020), the effect of participant age on participants’ overall evaluations of the robot in User Study 1 suggests that a more detailed consideration of different age groups might be worthwhile in future HRI research. Likewise, workers who are already used to be surrounded by robots in their working environment might highlight another perspective of HRI than our participants who were fairly inexperienced in using robots. In addition, User Study 1 and User Study 2 were conducted online which allowed us to reach large samples across countries. A further reason to opt for online studies was the fact that User Study 1 and User Study 2 were done before and right after the implementation and training processes of robot working memory. Therefore, online studies were considered safer for participants in this early stage of development. As a downside of online studies, however, experiences based on a video of the RB-KAIROS + robot might not convey a fully realistic experience of HRI. Considering the presented user studies as a part of an iterative research process, we will tie up with the present findings and extent them by overcoming some limitations of the present research. To do so, we will validate the present findings by conducting further interactive user studies taking place in a safe lab environment with lay participants and in an industrial setting with workers who are already experienced in using the RB-KAIROS + robot.

### 6.2 Contributions of the present research and conclusions

Our research approach to involve potential users into the implementation and training processes of robot working memory on an industrial RB-KAIROS + robot right from the beginning was clearly a novelty. It provided fruitful insights about participants’ ideas of robot memory. Likewise, participants’ precise suggestions for a safe, efficient, and comfortable human-aware robot navigation in the context of User Study 1 provide practical implications for developers of memory-based robot navigation. They are thus valuable for future research and development in HRI in industrial settings and beyond. The complementary use of quantitative measures and qualitative open-response formats and the consideration of participants’ attitudes toward robots, experiences, and demographics enabled a more holistic view on potential users and helped to better understand the psychological processes that might underlie HRI. We therefore strongly recommend to use quantitative and qualitative measures complementary and to consider participants’ attitudes, experiences, and demographics. In this regard, our measures of robot appearance, robot movements, and perceived robot memory that we provided in three languages, exhibited good reliabilities in both user studies which confirms their suitability for future HRI research. As such, besides practical implications for further research and development of human-aware robot navigation, our research provided new insights about participants’ concerns and preferences in HRI. At the same time, our findings raised new research questions and provided suitable approaches and measures to address them. This being provided, we encourage to involve potential users more strongly into future HRI research and development.

## Data Availability

The raw data supporting the conclusion of this article will be made available by the authors, without undue reservation.
